# Evidence of host adaptation in *Lawsonia intracellularis* infections

**DOI:** 10.1186/1297-9716-43-53

**Published:** 2012-06-20

**Authors:** Fabio A Vannucci, Nicola Pusterla, Samantha M Mapes, Connie Gebhart

**Affiliations:** 1 Department of Veterinary and Biomedical Sciences, College of Veterinary Medicine, University of Minnesota, St. Paul, MN, 55108, USA; 2 Department of Veterinary Medicine and Epidemiology, School of Veterinary Medicine, University of California, Davis, CA, 95616, USA

## Abstract

**Background:**

*Lawsonia intracellularis* is the causative agent of proliferative enteropathy, an endemic disease in pigs and an emerging concern in horses. Enterocyte hyperplasia is a common lesion in every case but there are differences regarding clinical and pathological presentations among affected species. We hypothesize that host susceptibility to *L. intracellularis* infection depends on the species of origin of the bacterial isolate. The objective of this study was to evaluate the susceptibilities of pigs and horses to *L. intracellularis* infection using either a porcine or an equine isolate.

**Materials and methods:**

Twelve foals and eighteen pigs were equally divided into three groups and infected with either a porcine or an equine isolate (10^9^*L. Intracellularis*/challenged animal), and a saline solution (negative control group). The animals were monitored regarding clinical signs, average of daily weight gain, fecal shedding of the bacteria by PCR and humoral serological response.

**Results:**

Foals infected with the equine isolate developed moderate to severe clinical signs and maintained a lower average of weight gain compared to control foals. Fecal quantitative PCR in equine isolate-infected foals revealed higher amounts of bacterial DNA associated with longer duration of shedding compared with porcine isolate-infected foals. All four foals infected with the equine isolate demonstrated higher IgG titers in the serum compared with porcine isolate-infected foals. In the pig trial, diarrhea and seroconversion were only observed in animals infected with the porcine isolate. Pathological changes typical of proliferative enteropathy were observed in the necropsied foal infected with equine isolate and in the two necropsied pigs infected with the porcine isolate.

**Conclusions:**

Evident clinical signs, longer periods of bacterial shedding and stronger serologic immune responses were observed in animals infected with species-specific isolates. These results show that host susceptibility is driven by the origin of the isolated *L. intracellularis* strain.

## Introduction

*Lawsonia intracellularis* is an obligate intracellular bacterium and the etiologic agent of proliferative enteropathy (PE), an intestinal hyperplasic disease characterized by thickening of the mucosa of the intestine due to enterocyte proliferation [1]. The disease has been reported in a variety of animal species, including nonhuman primates, wild mammals and ratite birds [2,3]. Since the 1990s, it has been endemic in pigs and one of the most economically important diseases in the swine industry [4]. In the last decade, the disease also has been frequently reported in weanling foals worldwide, and now is described as an emerging disease in the horse population [5-8].

Although hyperplasic lesions are present in every case of PE, there are some differences regarding clinical and pathological presentations among affected species. In pigs, there are two major clinical forms: a sporadic, acute, haemorrhagic diarrhea and a chronic, mild diarrhea [1]. A haemorrhagic form has also been reported in macaques but not in any other susceptible species [9]. Infected horses develop acute but non-haemorrhagic diarrhea. Furthermore, hypoproteinemia is an important clinical sign of PE in horses but it has not been reported in pigs. These observations demonstrate important host-specific characteristics of this infection.

Isolation and cultivation of *L. intracellularis* has only been achieved by using dividing cells in culture under strict microaerophilic conditions. These fastidious properties restrict opportunities to study the dynamics of inter-species transmission, potential reservoirs for the bacterium and host susceptibilities to different bacterial isolates. The disease has been experimentally reproduced in hamsters, pigs and horses using species-specific isolates or intestinal homogenate derived from infected animals [10-12]. Results from cross-species experimental infections in hamsters and mice models using intestinal homogenates or porcine *L. intracellularis* isolates have consistently reproduced subclinical disease and mild lesions in infected animals [13-15]. Therefore, the bacterium seems to adapt and persist differently depending on the species origin of the isolate. The susceptibility of pigs to equine isolates or vice-versa has not been reported and may provide relevant information about host adaptation or specificity of *L. intracellularis* infections.

We hypothesize that host adaptation to *L. intracellularis* infection is capable of driving the susceptibilities of pigs and horses depending on the species origin of the isolate. The objective of this study was to evaluate the susceptibilities of horses and pigs to *L. intracellularis* infection using porcine and equine isolates. The present study reports clinical signs, longer periods of fecal shedding of bacteria and stronger serologic immune responses in pigs and foals infected with their species-specific isolates.

## Materials and methods

### Challenge isolates and preparation

The present study used a porcine (PHE/MN1-00) and an equine (E40504) *L. intracellularis* strain isolated from a gilt and a foal, respectively, both affected with the acute form of PE. The pathogenicity of each of these isolates was previously established in a porcine and an equine experimental model [11,12]. Both strains were isolated and grown in murine fibroblast-like McCoy cells (ATCC CRL 1696), as described elsewhere [16,17]. Briefly, one-day-old McCoy cells growing in T_175_ cell culture flasks containing Dulbecco’s Modified Eagles Medium with 1% L-glutamine, 0.5% amphotericin B and 7% fetal bovine serum (FBS) were infected with 2 mL of *L. intracellularis* (with approximately 10^6^ organisms). Infected flasks were placed in an anaerobic jar, from which the atmospheric air was evacuated by a vacuum pump to 500 mmHg and replaced with hydrogen gas. The infected flasks were then placed in a Tri-gas incubator with 83.2% nitrogen gas, 8.8% carbon dioxide and 8% oxygen gas and incubated with a temperature of 37°C for seven days [18]. After a total of ten serial cell passages in vitro, the bacteria were pelleted, suspended in sucrose-potassium glutamate (pH 7.0; 0.218 M sucrose, 0.0038 M KH_2_PO_4_, 0.0072 M K_2_HPO_4_ and 0.0049 M potassium glutamate) solution with 10% FBS and stored at −80°C until the day of infection [16]. The inocula for both horse and pig experiments were identically prepared at the College of Veterinary Medicine of the University of Minnesota, using the same protocols for isolation and cultivation of *L. intracellularis.* For the horse trial, porcine and equine isolates were separately preserved in dry ice and shipped to the University of California-Davis Center for Equine Health. For both equine and porcine isolates the molecular identities were determined by multi-locus variable number tandem repeat (VNTR) analysis [19].

The number of *L. intracellularis* organisms was assessed by direct counting after immunoperoxidase staining of serial 10-fold dilutions prepared in sterile phosphate-buffered saline (PBS) using polyclonal *L. intracellularis*-specific antibody [16]. Additionally, quantitative PCR (qPCR) was performed using an aliquot from each inoculum in order to validate the direct counting and standardize the challenge doses used in the foal and pig experimental infection models [20].

### Animals

Twelve Quarter Horse foals between 4 and 5 months of age were randomly divided into three groups: Horse/Porcine isolate (*n* = 4); Horse/Equine isolate (*n* = 4) and Horse/Negative control (*n* = 4). Similarly, 18 Duroc-Landrace cross pigs at 3-weeks-of-age were allocated into three groups: Pig/Porcine isolate (*n* = 6); Pig/Equine isolate (*n* = 6) and Pig/Negative control (*n* = 6). The animals were obtained from a herd with no history of PE and each treatment group was housed in a different pen. Prior to study commencement, all animals were evaluated for any signs of illness by a full physical examination. In addition, blood and fecal samples from all animals were collected and tested for *L. intracellularis*-specific antibodies by immunoperoxidase monolayer assay (IPMA) and qPCR (described in section “Quantitative PCR and serological IgG response”) in order to document the negative status for each animal. Throughout the study, the foals were housed at the University of California-Davis Center for Equine Health and fed with free choice of grass and alfalfa hay and water and were supplemented daily with a commercial foal supplement. The pigs were housed in the isolation barns at the College of Veterinary Medicine of the University of Minnesota and fed with non-medicated nursery feed and water *ad libitum*. Horse and pig pens were cleaned once daily. All procedures were approved by the Institutional Animal Care and Use Committees of the University of California (horse study) and University of Minnesota (pig study). The horse and pig studies were conducted in the summer of 2010 and 2011, respectively.

### Experimental infection

On the challenge day (day 0), the frozen inoculum was thawed at 37°C and administered within 1 h of thawing. The foals were sedated with detomidine hydrochloride^a^ (0.01 mg/kg BWT) and inoculated with 50 mL of the inoculum via nasogastric intubation [12]. The pigs were restrained and inoculated with 30 mL of the inoculum using a stomach tube [16]. Control foals and pigs received sucrose–potassium–glutamate solution by the same route of inoculation.

### Monitoring and sample collection

Monitoring and sample collection were conducted similarly in the horse and pig studies. All animals were observed daily for general attitude and appetite for 56 days post-inoculation (pi). Once weekly, the weight of each animal was recorded in order to determine average daily weight gains throughout the study period. Feces were collected directly from the rectum every other day and submitted for qPCR [20]. Blood samples were collected on days 0, 7, 14, 21, 28, 42 and 56 pi to determine serum concentration of total solids and for serologic analysis by IPMA [21].

Due to the non-terminal design of the horse study, foals developing either moderate to severe acute (fever, depression, anorexia, colic, diarrhea) or chronic (weight loss, peripheral edema) signs and/or hypoproteinemia (<5.0 g/dL) were treated with doxycycline hyclate^b^ (10 mg/kg, PO, q 12 h) for 10 days. Additional supportive treatment including intravenous flunixine meglumine^c^ (1.1 mg/kg, IV, q 12 h) and replacement fluids^d^ (4–6 mL, IV, q 1 h) were given based on the patient’s need.

### Quantitative PCR and serological IgG response

All fecal samples were analyzed by qPCR for *L. intracellularis* DNA at the School of Veterinary Medicine of the University of California-Davis, as described elsewhere [20]. Briefly, DNA purification was performed using an automated nucleic acid extraction system^e^, according to the manufacturer’s recommendations. Absolute quantification was calculated using a standard curve for *L. intracellularis* and expressed as copy numbers of the *aspA* gene of *L. intracellularis* per gram of feces. The standard curve was determined by using 10-fold dilutions of *L. intracellularis* derived from cell culture in McCoy cells added to *L. intracellularis*-free equine feces. Furthermore, a qPCR assay targeting a universal sequence of the bacterial 16S rRNA gene was used as quality control (i.e. efficiency of DNA purification and amplification) and as an indicator of fecal inhibition [22].

Blood samples for the collection of serum were drawn from all animals in order to determine the concentration of total solids using a refractometer and to measure anti-*L. intracellularis* specific IgG by IPMA at the College of Veterinary Medicine of the University of Minnesota, as previously reported [21]. Positive serum samples (titer ≥ 60) were tested to endpoint dilution and titers were reported as the reciprocal of the dilution.

### Post-mortem examination, histology and immunohistochemistry

Two pigs from each group were euthanized on day 21 pi and evaluated for typical PE lesions. Intestinal samples from jejunum, ileum, cecum and colon were collected, fixed in 10% buffered formalin, processed routinely for histology, embedded in paraffin, and sectioned 5 μm thick. Two sections were prepared: one section was stained by haematoxylin and eosin [23] and the other by immunohistochemistry (IHC) using the streptavidin method with polyclonal antibodies to *L. intracellularis*[24]. The level of infection was assessed by IHC based on the amount of positive labeled antigen present in the intestinal sections: Grade 0 = no positive antigen labeled; Grade 1 = one isolated focal area of antigen labeled; Grade 2 = multi-focal areas of antigen labeled; Grade 3 = majority of the mucosa has positive antigen labeled; and Grade 4 = all of the mucosa has positive antigen labeled [11]. Histology and IHC procedures were conducted in the Veterinary Diagnostic Laboratory of the University of Minnesota.

### Statistical analysis

Descriptive analyses were used to describe clinical findings among the different groups due to the limited number of animals. Statistical analysis was performed by use of Wilcoxon–Mann–Whitney tests to assess differences in daily weight gain among the different groups. The area under curve (AUC) for the amount and duration of fecal shedding, as well as for the magnitude and duration of measureable IgG titers against *L. intracellularis*, were calculated using trapezoid rule in the SAS software (version 9.2). The Wilcoxon–Mann–Whitney test was used to compare the experimental groups based on AUC. Statistical significance was defined at values of *p* < 0.05.

## Results

### Experimental infection and pathological findings

The challenge doses were standardized for the horse and pig trials in order to avoid any influence of dose effect on the clinical, pathological or immune response. Based on the quantification of *L. intracellularis* organisms (described in section “Challenge isolates and preparation”), which was confirmed by qPCR, each animal received 10^9^ bacteria intragastrically. In addition, the molecular identities of the porcine and equine isolates were confirmed after the experimental infection by VNTR typing using PCR positive samples.

**Figure 1:**
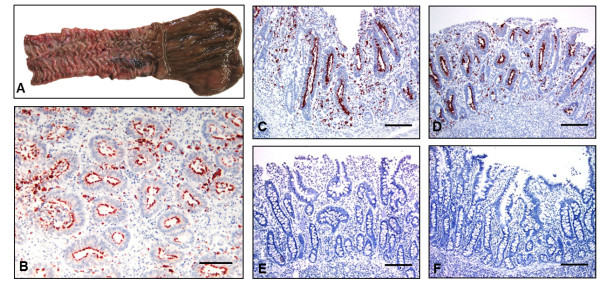
**Pathological findings. A**) Terminal ileum. Equine isolate-infected foal euthanized on day 24 pi. Hyperaemia associated with diffuse and severe thickening of the intestinal mucosa compatible with PE. **B**) Histologic section of ileum. Equine isolate-infected foal euthanized 24 days pi. *L. intracellularis* antigen-specific staining with AEC substrate-chromogen and counterstained with Mayer’s hematoxylin. Diffuse hyperplasia of immature enterocytes and severe reduction in the number of goblet cells associated with the diffuse presence of intracellular bacteria in the apical cytoplasm. Scale bar, 100 μm. **C–D**) IHC staining. Ileal mucosa of porcine isolate-infected pigs euthanized 21 days pi. Diffuse presence of the bacteria in the apical cytoplasm of the hyperplastic enterocytes and in the lamina propria. Scale bar, 200 μm. **E–F**) IHC staining. Ileal mucosa of equine isolate-infected pigs euthanized 21 days pi. Absence of hyperplastic enterocytes or *L. intracellularis* antigen associated with diffuse presence of goblet cells in the intestinal epithelium. Scale bar, 200 μm.

Diarrhea and significant lower daily weight gain (*p* < 0.05) were observed in pigs infected with the porcine isolate and in foals infected with the equine isolate, as shown in Table 1. Three equine isolate-infected foals developed moderate to severe clinical signs typical of equine PE, including depression, anorexia, colic and peripheral edema. Hypoproteinemia (<5.0 g/dL) was also observed in these foals (ranging from 4.1 to 4.7 g/dL) between 21 and 28 days pi. Hypoproteinemia was not observed in any infected pigs. Because of the severe clinical signs, one equine isolate-infected foal was treated according to the protocol previously described, but it did not respond to antimicrobial and supportive treatment and it was then humanely euthanized 24 days pi. A full necropsy revealed severe and diffuse (from duodenum to cecum) thickening of the intestinal mucosa associated with the presence of large numbers of intracellular *Lawsonia*-specific antigen identified by IHC (Figure 1a and b). Based on the typical clinical signs of PE, the peak of the experimental infection occurred between the third and fourth week pi in both pigs and foals. Foals infected with the porcine isolate, pigs infected with the equine isolate and negative control groups failed to show clinical signs, hypoproteinemia (foals), lower average of weight gain or pathological changes.

**Table 1:** Average of daily weight gain (mean ± standard error) throughout the entire study period

**Infected species**		**L. intracellularis isolate**	
	Control	Porcine	Equine
Pig (*n* = 6)	0.59 ± 0.09 ^a^	0.32 ± 0.07 ^b^	0.54 ± 0.11 ^a^
Horse (*n* = 4)	0.90 ± 0.04 ^a^	0.75 ± 0.04 ^a^	−0.07 ± 0.28 ^b^

Pairwise comparisons are within columns: means that do not share letters are significantly different (*p* < 0.05).

The experimental infection in pigs revealed macroscopic and histologic lesions typical of porcine PE in the two animals infected with porcine isolate and euthanized 21 days pi. These lesions were associated with the presence of *Lawsonia*-specific antigen in the intestinal epithelium (Figure 1c and d). Neither the two pigs infected with the equine isolate nor the two negative controls which were euthanized 21 days pi showed any clinical or pathological changes (Figure 1e and f).

### Quantification of fecal *L. intracellularis* DNA

**Figure 2:**
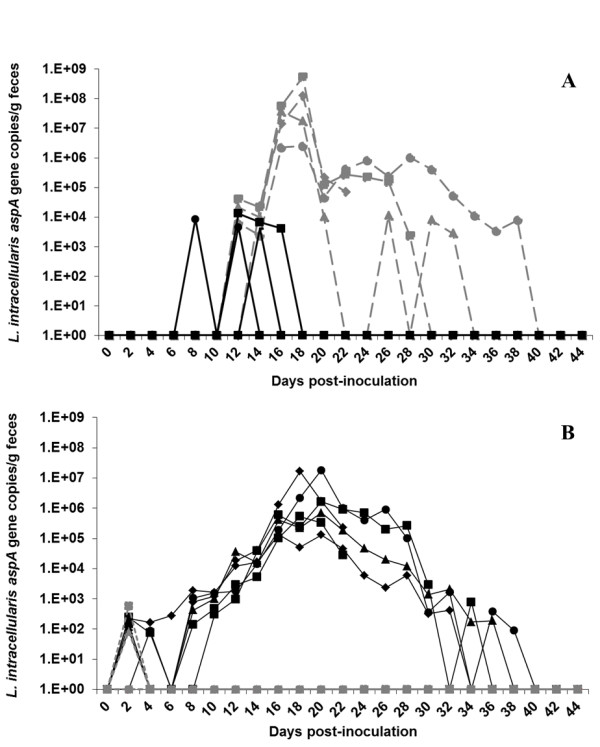
**Quantification of the *L. intracellularis* DNA in the feces performed by qPCR.** The results are expressed as copy numbers of *aspA* gene copies of *L. intracellularis* per gram of feces throughout the study period. **A**) Foal experiment. **B**) Pig experiment. Black-solid lines represent animals infected with the porcine isolate and grey-dashed lines represent animals infected with the equine isolate.

Results of fecal shedding of *L. intracellularis* throughout the study period are summarized in Figure 2. Positive PCR signals for the universal bacterial 16S rRNA gene were detected in all fecal samples demonstrating the efficiency of the DNA extraction protocol. *L. intracellularis* DNA was observed in the feces of foals infected with the equine isolate from 12 to 38 days pi. Three foals infected with the porcine isolate shed bacteria. However, *L. intracellularis* DNA was detected in these animals at only four time points and no more than 10^4^ bacterial organisms per gram of feces was detected (Figure 2a). The mean areas under the “time – *L. intracellularis* organisms/g” curve (Figure 2a) were significantly different (*p* < 0.05) between foals infected with equine and porcine isolates. As a result, equine isolate-infected foals revealed higher amounts of bacterial DNA in the feces associated with longer duration of shedding compared with porcine isolate-infected foals.

In the pig trial, animals infected with the porcine isolate showed higher and longer shedding of bacteria in the feces throughout the study period (*p* < 0.05) (Figure 2b). In these animals, PCR detection of *L*. *intracellularis* lasted from day 2 to 38 pi. Two pigs infected with the equine isolate shed the bacteria at low levels (10^2^ bacteria/g of feces) on day 2 pi. All foals and pigs in the negative control group remained negative for the entire study period.

### Serological IgG response

**Figure 3:**
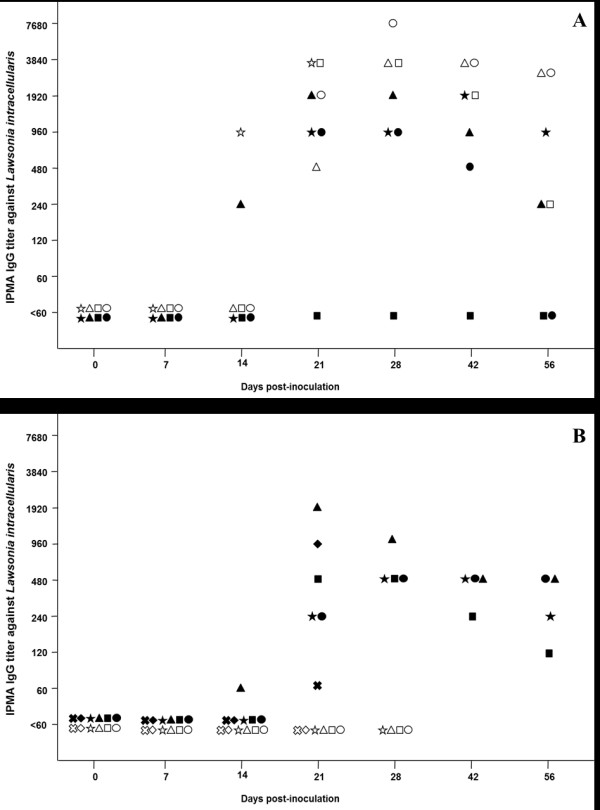
**Serologic response against *L. intracellularis* throughout the study period. A**) Foal experiment. **B**) Pig experiment. White points represent animals infected with the equine isolate and black points represent animals infected with the porcine isolate.

All four equine isolate-infected foals demonstrated higher IgG titers (≥3840) against *L. intracellularis* in the serum compared with the porcine isolate-infected foals (≤1920) (Figure 3a). One foal infected with the porcine isolate showed no measurable serologic response during the entire study period. The mean areas under the time—titer curve of the IPMA titers were not significantly different (*p* < 0.05) between foals infected with equine and porcine isolates. However, because there was no serum from day 28 pi in the foal euthanized on day 21, the area under the curve for this animal was underestimated in the analysis. Simulating IgG titers of at least 960 on days 28 and 42 for this foal, significant difference (*p* < 0.03) would be observed in the time—titer curve of the IPMA between foals infected with porcine and equine isolates.

The serologic responses in the pig experiment are summarized in Figure 3. There was no detectable serologic response in pigs infected with the equine isolate at any time point. On the other hand, the majority of porcine isolate-infected pigs demonstrated IgG titers (> 120) against *L. intracellularis* from day 21 pi. The serological responses were persistent throughout the study in foals and pigs infected with species-specific isolates. However, foals infected with the equine isolate had much stronger immune responses (*p* = 0.057) than those pigs infected with the porcine isolate.

## Discussion

Marked clinical signs and pathological changes typical of PE associated with longer periods of bacterial shedding and stronger serologic immune responses were observed in pigs and foals infected with species-specific isolates. These observations were made despite the identical doses of *L. intracellularis* used in the horse and pig experimental infections and support the hypothesis that host susceptibilities are driven by the origin of the isolated strain. Pigs and foals demonstrated a similar pattern of fecal shedding, which lasted until 39 days pi, but the bacterial DNA was identified earlier in pigs (day 2 pi) compared to foals (day 8 pi). In those animals infected with species-specific isolates, foals exhibited a more robust serological IgG response compared with pigs which may reflect a characteristic related to the host response, regardless of the *L. intracellularis* isolate. Additionally, hypoproteinemia was an important sign of PE in the affected horses. Despite the phenotypic differences regarding the susceptibilities of these two species, gross and histological lesions characterized by thickening of intestinal mucosa due to proliferation of enterocytes were identical in those animals infected with species-specific isolates.

PE has been previously reproduced using pure cultures or intestinal mucosa homogenates derived from infected animals [11,12,25]. Since *L. intracellularis* is a fastidious organism and extremely difficult to isolate and propagate, mucosal homogenate challenge models have been reported in pigs using intestinal mucosa from PE-affected pigs [26-28]. Hamsters and mice infected with intestinal homogenates from PE-affected pigs have consistently developed subclinical PE associated with mild lesions, mainly found in the cecum and colon [15,29]. However, dehydration and profuse diarrhea were observed in hamsters inoculated with intestinal homogenate or filtrate derived from PE–affected hamsters [10,25]. Studies using mice experimentally infected with pure cultures of porcine *L. intracellularis* isolates have revealed inconsistent results. Smith et al. [14] described pathological changes in wild-type 129-Sv-Ev and IFN-γ receptor knockout (IFN-γ R^-^) mice infected with a porcine isolate. Go et al. [30] reported gross and histological lesions in IFN-γ R^-^ mice but not in three wild-type mice strains (ICR, BALB/c and C57BL/6). A more recent study showed mild histological lesions in four mice strains, including BALB/c and C57BL/6 [31]. As described above, the lack of availability of *L. intracellularis* isolates from different host species has limited the experimental infection studies to the use of porcine isolates. Recently, our research group isolated a new equine strain and its virulence was confirmed in a foal experimental model [12]. This allowed us to perform the present study which is the first cross-species experimental model using pure cultures of *L. intracellularis* and to evaluate different host susceptibilities and adaptation to species-specific bacterial isolates.

The challenge models previously described have contributed to advances in the prevention and control of PE by determining the efficacy of different antimicrobial drugs and vaccination protocols [16,27,32]. Nevertheless, little is known about the epidemiology and ecology of *L. intracellularis* infection, especially regarding inter-species transmissions, source or reservoir hosts of *L. intracellularis* and potential biological or mechanical vectors. Based on the pattern of bacterial shedding (Figure 2), foals infected with the porcine isolate and pigs infected with the equine isolate showed low levels of *L. intracellularis* DNA in the feces during a short period of time at the earlier stages of the experimental infection. Based on these findings and the uncommon opportunity of pigs and foals to share the same environment in the modern pig production system or horse breeding farms, any direct cross-species transmission in the field between these two species is unlikely.

Free-living animals have been speculated to be a reservoir of PE by allowing the maintenance of *L. intracellularis* in the wild population with successive introductions into domestic pig or horse farms. Pusterla et al. [20] detected bacterial DNA by fecal PCR in a variety of domestic and wild species, including dogs, cats, mice, rabbits, opossums, skunks and coyote, on horse breeding farms with documented occurrence of EPE. The involvement of wild animals as biological vectors for *L. intracellularis* in the pig production system has also been investigated. Using quantitative PCR, Collins et al. [33] recently identified rats trapped in endemic pig farms shedding less than 10^5^*L. intracellularis*/g of feces. However, these authors also reported that a small proportion of rats shed more than 10^8^ bacteria per gram of feces. Associating these observations with the results from our study leads us to speculate on the presence of different sources or reservoir species for *L. intracellularis* infections in horses and pigs. In addition, the lack of evidence supporting inter-species transmission between horses and pigs helps confirm the host adaptation to species-specific isolates demonstrated in the present study.

In order to support these hypotheses, the phenotypic observations described in the present study may be linked with genotypic differences between *L. intracellularis* isolates. Comparison of the 16S ribosomal DNA sequences showed a high degree of similarity among *L. intracellularis* from pigs, hamsters, deer and ostriches [34]. Four sets of primers targeting hypervariable regions of the *L. intracellularis* genome were used in the development of the VNTR technique [19]. This technique has shown unique and distinct VNTR profiles from epidemiologically unrelated outbreaks in pigs and horses and it was performed to confirm the molecular identity of the porcine and equine isolates used in the present study. A comprehensive analysis involving whole-genome sequencing of equine, as well as porcine, *L. intracellularis* isolates is crucial to identify any genomic variations associated with different host adaptations and susceptibilities.

In conclusion, the present study demonstrated marked clinical signs, longer periods of bacterial shedding and stronger serologic immune responses in foals and pigs experimentally infected with their species-specific isolates. This evidence of host adaptation in these species suggests that host susceptibilities to PE are driven by the origin of the *L. intracellularis* isolate. Comparative genomic analysis is a promising route to associate phenotypic characteristics with potential genomic variations between porcine and equine isolates and may help to characterize species-specific *L. intracellularis* strains and potentially novel bacterial subspecies or genotypes.

## Endnotes

^a^ Dormosedan, Pfizer Animal Health, Exton, Pa.

^b^ Doxycycline, Reva Pharmaceuticals, Sellersville, Pa. 

^c^ Banamine, Schering-Plough Animal Health, Union, Nj.

^d^ Plasma-Lyte A, Baxter Healthcare Corporation, Deerfield, Il. 

^e^ CAS-1820 X-tractor Gene, Corbett Life Science, Sydney, Australia.

## Abbreviations

PE, Proliferative enteropathy; FBS, Fetal bovine serum; VNTR, Variable number tandem repeat; PBS, Phosphate-buffered saline; IPMA, Immunoperoxidase monolayer assay; pi, Post-inoculation; qPCR, Quantitative PCR; IHC, Immunohistochemistry; AUC, Area under curve; IFN-γ R-, IFN-γ receptor knockout.

## Competing interests

The authors declare they have no competing interests.

## Authors’ contributions

Conceived and designed the experiments: FAV, NP, CG. Performed the experiments and analyzed the data: FAV, NP, SMM, CG. Wrote the paper: FAV, NP. Coordinated and helped to draft the manuscript: CG. All authors read and approved the final manuscript.
